# Reducing NICU ventilator days by preventing fluid overload with the CAN-U-P-LOTS standardized bundle

**DOI:** 10.1038/s41390-025-04078-x

**Published:** 2025-07-11

**Authors:** David J. Askenazi, Lindsey Gordon, Russell Griffin, Monica Collins, Allison Black, Namasivayam Ambalavanan, Tennille Webb, Meggie Mathis, Kara Short, Alyssa Umberger, Colm Travers

**Affiliations:** 1 Division of Nephrology, Department of Pediatrics, University of Alabama at Birmingham (UAB), Birmingham, AL USA; 2 Department of Epidemiology, UAB, Birmingham, AL USA; 3 Division of Neonatology, Department of Pediatrics, UAB, Birmingham, AL USA; 4 Children’s of Alabama, Birmingham, AL USA

## Abstract

**Background:**

Fluid overload in critically ill neonates and infants is associated with higher ventilation days, prolonged length of stay, and mortality.

**Methods:**

This quality improvement study enrolled infants admitted to Children’s of Alabama NICU (excluding those with tracheostomies, severe congenital kidney or heart disease, DNR status, or severe genetic conditions). We compared 7 months of pre-intervention data (211 neonates) with 7 months of post-implementation data (218 neonates). Bundle implementation for at least 5 days occurred for sepsis, spontaneous intestinal perforation, necrotizing enterocolitis, acute kidney injury, positive fluid balance >10%, hypotension, and major surgeries. The primary hypothesis was that the unit-wide ventilator-free days would increase after bundle implementation.

**Results:**

We found special cause variation with an increase in the percentage of ventilator-free and oxygen-free days coinciding with bundle introduction. The ventilator-free days were higher in the post-era compared to the pre-era (5592/8335 (67%) vs. (3732/6619) (56%); *p* < 0.001). Oxygen-free days and NICU length of stay showed similar findings.

**Conclusions:**

Implementation of a fluid overload prevention bundle was associated with increased ventilator-free days, oxygen-free days, and shortened NICU duration. Additional studies are needed to better understand these associations and externally validate our hypothesis in other populations.

**Impact:**

- Fluid overload leads to poor clinical outcomes, including the need for ventilatory support.
- Prolonged ventilation has a deleterious effect on the lungs due to barotrauma and leads to complications (i.e., pneumonia), longer length of stay, and increased costs.
- After consensus from a multi-disciplinary team, we implemented a strategy using the CAN-U-P-LOTS bundle designed to prevent fluid overload in critically ill infants.
- We showed an increase in the number of ventilator-free days, oxygen-free days, and shorter length of stay.
- Studies are needed to validate our single-center study.

## Introduction

Fluid overload, a pathologic state of positive fluid balance associated with clinically observed events,^1^ in neonates is associated with poor clinical outcomes, including acute kidney injury (AKI), heart failure, necrotizing enterocolitis (NEC), intraventricular hemorrhage, and mortality.^2–7^ Additionally, a positive fluid balance early in the first postnatal weeks is associated with bronchopulmonary dysplasia^6,8,9^ and increased duration of mechanical ventilation in neonates, pediatric patients, and adults.^7,8,10,11^ The AWAKEN (Assessment of Worldwide Acute Kidney injury Epidemiology in Neonates) study, a 24-center observational cohort study, demonstrated that critically ill preterm and near-term/term neonates with negative fluid balance were less likely to require mechanical ventilation at postnatal day 7.^12,13^ In 2020, Matsushita et al. showed that extremely low birth weight infants with positive fluid balance had higher rates of death, mean airway pressure and longer duration of mechanical ventilation.^14^

In single-center studies of critically ill children admitted to pediatric intensive care units, intervention bundles designed to reduce fluid overload have been successful.^15–17^ For example, Charaya et al. in 2025^18^ and Diaz et al. in 2018^15^ showed that bundles designed to reduce the peak fluid overload in ventilated critically ill children are associated with a lower duration of mechanical ventilation and length of stay. In adults, fluid overload prevention bundles are associated with a decreased rate and severity of AKI, need for dialysis, length of stay, and major adverse kidney events at 30, 60, and 90 days.^17,19^ Whether this approach could improve clinical outcomes in critically ill neonates and infants admitted to the neonatal intensive care unit (NICU) has yet to be explored.

In 2022, we reviewed the medical literature on fluid overload in neonates and proposed the CAN-U-P-LOTS intervention bundle (Table 1) designed to mitigate fluid overload in critically ill neonates and infants.^8^ Each bundle element pairs an assessment with an intervention designed to prevent a positive fluid balance by optimizing urine output or reducing fluid intake. In September 2023, we instituted a unit-wide bundle designed to standardize care early during critical illness in high-risk neonates admitted to the Children’s of Alabama NICU following consensus approval from both nephrology and neonatology. In order to understand the impact of bundle implementation in our unit, we performed a quality improvement study. Our primary hypothesis was that implementation of the CAN-U-P-LOTS bundle in at-risk patients would increase ventilator-free days by 20% within 6 months across the NICU. Secondary outcomes included increased oxygen-free days, decreased NICU days, and decreased mortality.

**Table 1:** CAN-U-P-LOTS bundle to prevent and treat fluid overload

BUNDLE ELEMENT	INTERVENTION
**C** – Cause/Compartment syndrome	Consider and address the underlying cause of fluid overload if present
	- If significant abdominal distension consider measuring bladder pressure, and if bladder pressure is elevated (>10–12 mmHg) and oliguria is present, consider abdominal tap/drain
**A** – Albumin	Assess for hypoalbuminemia. If albumin <2 g/dL, give 1 g/kg of 25% intermittently until albumin >2 g/dl. (If extremely low birth weight in the first few weeks of life, consider 1 g/kg 25% albumin if <1.5 g/dL)
**N** – Nephrotoxic medications	Review the medication list for nephrotoxic medications and transition to alternatives as feasible
	Avoid additional nephrotoxic medications if possible
	If using Vancomycin or aminoglycosides, obtain the trough level and ensure that levels are below the recommended threshold before redosing
**U** – Uric acid	Obtain uric acid level. If >9 mg/dL, screen for G6PD and if negative, give Rasburicase 0.1 mg/kg
**U** – Ultrafiltration	Consider ultrafiltrate (i.e., PD, SCUF, or CRRT) if unable to meet desired fluid balance goals or unable to provide nutrition without worsening fluid overload
**P** – Perfusion	Incrementally increase mean arterial pressure (MAP) goals by 5–10 mmHg q 4 h until an increase in UOP is achieved. Do not exceed the 90^th^ percentile for age.
	Consider increasing intravascular volume with 2 × 10 mL/kg of packed red blood cells to keep HCT > 30–50%, or FFP or crystalloid
	Use vasopressors to maintain the goal if no response to fluids
	Consider the addition of hydrocortisone for refractory hypotension
**L** – Lasix stress test	If, after optimizing intravascular volume and MAP, the patient remains oliguric (<1 mL/kg/h), consider the Lasix stress test
	Give 1 mg/kg Lasix, document UOP within 2 h of administration
	- If responsive (>1 ml/kg/h), assure adequate perfusion. Can adjust diuretics to maintain fluid goals
	- If non-responsive, avoid escalating diuretic use and call nephrology
**O** –Output/obstruction	Measure urine output continuously with an indwelling or external urine collection device
	- If concern for inadequate bladder drainage, obtain an ultrasound
	- If bladder obstruction is confirmed or suspected, place an indwelling bladder catheter
**T** – Total fluid intake	Concentrate fluids while maintaining proper nutrition
	Document total fluid intake goals
**S** – Steroids	Consider the addition of hydrocortisone for refractory hypotension if requiring pressors

## Methods

### Context

This study was performed as part of a single-center, quality improvement initiative for critically ill infants at Children’s of Alabama (CoA), utilizing to SQUIRE 2.0 guidelines.^20^ CoA is a free-standing children’s hospital with a 54-bed, level IV NICU. There are approximately 210 staff nurses, 22 neonatology and 7 pediatric nephrology faculty members, 9 neonatology and 3 pediatric nephrology fellows, 30 neonatology NPs and 2 acute nephrology NPs, and 2 NICU-dedicated pharmacists.

For this pre- and post-implementation quality improvement study, the monthly outcome data were pooled and tracked on run charts. We excluded patients with the following conditions because the conditions would affect the primary outcome: those with tracheostomies, congenital kidney failure requiring dialysis within the first postnatal week, congenital heart failure requiring surgery in the first postnatal week, life-limiting chromosomal anomalies not expected to live past two years of age, and intention to withdraw life sustaining therapy. Patients who were on extra-corporeal membrane oxygenation and who did not meet exclusion criteria were included.

### Planning the intervention

The clinical bundle was initially proposed in a review article by Weaver et al. entitled “Neonatal fluid overload – ignorance is no longer bliss”.^8^ This review was performed in collaboration between members of the division of neonatology and pediatric nephrology at The University of Alabama at Birmingham. Not only did the review summarize studies on the negative impact of fluid overload in neonates, but it also outlined the rationale and evidence for interventions designed to prevent and mitigate fluid overload. The proposed CAN-U-P-LOTS bundle (outlined in Table 1) paired specific assessments with interventions. The IRB at the University of Alabama at Birmingham reviewed and approved the quality improvement project under a Not Human Subjects Research (NHSR) designation.

All providers and NPs received bundle education between August 1 and August 31, 2023. We held three comprehensive learning sessions with the neonatal NP and one with the neonatology attendings/fellows to review studies on neonatal fluid overload, the CAN-U-P-LOTS intervention bundle, criteria for entering and exiting the bundle, data tracking, and pre-determined objectives. We developed a common slide deck and distributed it to all attendings, fellows, and NPs in the neonatology and nephrology divisions. We provided badge cards to all providers identifying aspects of the mnemonic, inclusion/exclusion criteria, and the algorithm that enables patients to exit the bundle. We placed a laminated copy of the inclusion criteria at each NICU bedside for registered, bedside nurses to help identify patients for bundle enrollment.

### Intervention

After the education of all providers, the bundle was implemented in September 2023. All neonates admitted to the NICU at Children’s of Alabama were screened daily to see if they met the inclusion criteria for the bundle. irrespective of gestational age, birth weight, or current weight. To be included in the clinical bundle, the patient had to meet one or more of the following criteria:

- Sepsis is defined as antibiotic use for >72 h.
- New-onset hypotension requiring pressor support.
- Major surgeries (omphalocele/gastroschisis repair, laparotomy, bowel resection, anorectal malformation repair, congenital diaphragmatic hernia, and tracheoesophageal fistula).
- ≥Stage 2 AKI defined by serum creatinine KDIGO criteria (doubling of serum creatinine (SCr) from baseline).

- Baseline SCr was defined as the lowest previous SCr (excluding those measured prior to 48 h postnatally as previously done in recent neonatal AKI studies^21–28^).

- Clinical edema in association with >10% fluid balance positive^29^ is defined as:

- $${{{\rm{Fluid\; balance}}}}=\frac{{Current\; weight}-{estimated\; dry\; weight}}{{Estimated\; dry\; weight}}x100$$

The baseline weight was defined as the weight prior to the patient becoming ill. Weights were encouraged to be done daily, but were left to the clinical team based on the clinical scenario.

- Necrotizing enterocolitis: ≥Stage 2 modified Bell’s criteria
- Spontaneous intestinal perforation (SIP) without proven NEC

**Fig. 1:**
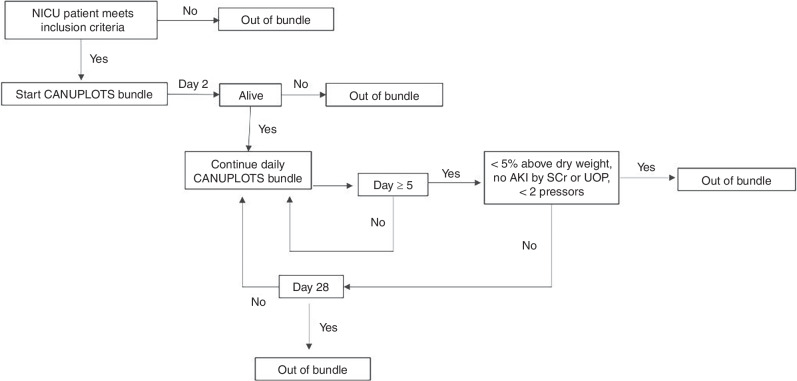
Algorithm for entering and exiting the CAN-U-P-LOTS bundle. Patients meet at least one criterion to enter the bundle. They must meet all 3 criteria (<5% above dry weight, no AKI, and <2 pressors) to come out of the bundle.

Patients who met bundle inclusion criteria remained in the bundle for at least 5 days. The algorithm outlined in Fig. 1 was used to determine when a neonate could exit the daily bundle. Patients could exit the bundle any time after 5 days if the patient met all three of the following criteria: (1) at their dry weight, (2) on less than two pressors for blood pressure support, and (3) without stage 2 AKI by SCr. Patients remained in the bundle for a maximum of 28 days.

While the patient was in the bundle, the clinical team worked through the assessment and interventions for each element of the CAN-U-P-LOTS bundle on a daily basis. Importantly, the bundle elements are recommendations, and clinical decisions were made on an individual basis at the discretion of the clinical team. At our institution, the practice of starting hydrocortisone for hypotension is individualized, but typically, vasopressor support is initiated before hydrocortisone.

During this time, the nephrology NP (KS and AU) and nephrology fellow (LG) reviewed workflow and recommendations with the rounding team periodically. In the middle of the intervention period, the nephrology fellow/NP performed formal re-education with the NICU NPs in person to review the bundle elements and the quality improvement. For NP who were not able to attend in person, email communication with a PowerPoint presentation was distributed. The quality improvement data was not shared with the NICU team until after the conclusion of the study.

### Data abstraction

Bundle information was collected through paper checklists designed to track patients and interventions (i.e., reasons for bundle, elements of the bundle) (Appendix 1). This form was filled out by the primary NP daily during rounds. If elements were not entered, the nephrology NP/Fellow assisted in data collection through evaluation from the EMR. With the checklist elements in hand, the clinical research nurse (JP) transcribed the data into Excel sheets.

We evaluated data from patients admitted prior to the intervention (January 1 to August 1, 2023) vs. after the bundle implementation (September 1, 2023, to April 30, 2024). Pre-intervention, baseline data were obtained retrospectively. Post-intervention data were collected through evaluation electronic medical record and abstracted from information routinely collected for quality improvement efforts (i.e., NICU admission and discharge dates, birth weight, gestational age, sex, and race). Respiratory support information was obtained from an ongoing quality improvement database that focuses on ventilator support for all children admitted to the NICU.

### Outcomes

The primary outcome was the percentage of ventilator-free days for the unit, which were calculated by the total days of ventilator use divided by the number of patient days in the entire unit for a given month. Secondary outcomes included oxygen-free days, NICU length of stay, and mortality.

### Statistical analysis

Demographic and clinical characteristics were compared between the pre- and post-bundle implementation eras using a *χ*^2^ or Wilcoxon rank sum test for categorical and continuous variables, respectively.

Data for the primary outcome were tracked on U-charts as the percentage of ventilator (or oxygen) free days divided by NICU days. Assessment for special cause variation was performed. Special cause variation in quality improvement reflects a significant change outside of the control limits. We determined special cause variation according to standardized rules for U-chart interpretation using the provided 2-sigma and 3-sigma confidence limits, which include: (a) any single data point outside control limits, (b) 8 consecutive points above or below the mean line, and (c) ≥6 consecutive points all moving in the same direction.^30^

Next, to compare the proportion of total days that were ventilator-free or oxygen-free between eras, we performed a negative binomial regression with patient days for a given month as the model offset. Within each era, monthly percent changes (MPCs) were estimated from an interaction of continuous month number within the era and era type (i.e., pre- or post-implementation). All analyses were performed using SAS v9.4, and *p*-values < 0.05 were considered statistically significant.

## Results

**Fig. 2:**
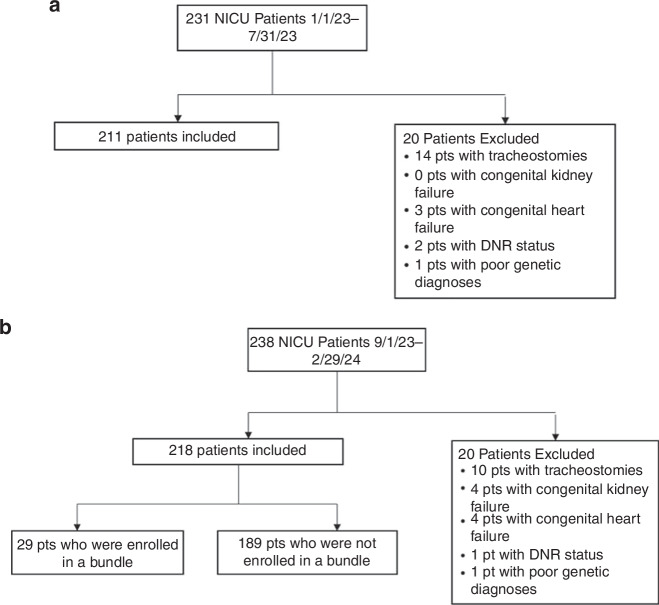
Consort diagram for the study. **a** Pre-intervention and **b** post-intervention.

Figure 2 is the consort diagram for each era. In the pre-intervention era, 211/231 (91%) were evaluated, as 20/231 (9%) met exclusion criteria (14 tracheostomies, 3 congenital heart failure, 2 DNR status, and 1 poor genetic diagnosis). In the post-intervention era, 218/238 (91%) were followed as 20/238 (9%) met exclusion criteria (10 tracheostomies, 4 congenital kidney failure, 4 congenital heart failure, 1 DNR status, and 1 poor genetic diagnosis).

A comparison of the demographics by era is described in Table 2. There were 53.8% males and 45.9% females. The majority of neonates were Caucasian (50.2%) or Black (37.4%), with fewer Hispanic (11%) or Asian (0.5%) patients. The most common birth weight category was >2500 g (48.3%), followed by 1501–2500 g (18.4%), and <750 g (15.3%), with 1001–1500 g (10.8%) and 751–100 g (7.2%) being less common. The most common GA category was >37 weeks (43.1%). The remainder were 32–37 weeks (23.5%), <28 weeks (21.4%), and least commonly 28–32 weeks (11.9%). We did not find statistically significant differences between any of these demographics by era (all *p*-values > 0.4).

**Table 2:** Comparison of demographic and clinical outcome data by period of bundle implementation

	Total *N* = 429	Pre-bundle *N* = 211	Post-bundle *N* = 218	*p*-value*
**DEMOGRAPHIC**				
Sex, *n* (%)				
Male	231 (53.8)	117 (55.5)	114 (52.3)	0.46
Female	197 (45.9)	93 (44.1)	104 (47.7)	
Indeterminate	1 (0.2)	1 (0.5)	0 (0.0)	
Race, *n* (%)				
Asian	2 (0.5)	2 (1.0)	0 (0.0)	0.49
Black	160 (37.4)	160 (37.4)	80 (36.7)	
Caucasian	215 (50.2)	215 (50.2)	113 (51.8)	
Hispanic	47 (11.0)	23 (11.0)	24 (11.0)	
Unknown	4 (0.9)	3 (1.4)	1 (0.5)	
Birth Weight, *n* (%)				
<750 g	64 (15.3)	29 (14.1)	35 (16.4)	0.75
751–1000 g	30 (7.2)	18 (8.8)	12 (5.6)	
1001–1500 g	45 (10.8)	23 (11.2)	22 (10.3)	
1501–2500 g	77 (18.4)	38 (18.5)	39 (18.3)	
>2500 g	202 (48.3)	97 (47.3)	105 (49.3)	
Gestational Age, *n* (%)				
<28 weeks	92 (21.4)	49 (23.2)	43 (19.7)	0.71
28.1–32 weeks	51 (11.9)	22 (10.4)	29 (13.3)	
32.1–37 weeks	101 (23.5)	50 (23.7)	51 (23.4)	
>37.1 weeks	185 (43.1)	90 (42.7)	95 (43.6)	
**CLINICAL**				
NICU disposition, *n* (%)				
To home or medical foster care	169 (39.4)	90 (42.7)	79 (36.2)	0.35
Transferred out of the NICU	216 (50.3)	99 (46.9)	117 (53.7)	
Died	44 (10.3)	22 (10.4)	22 (10.1)	
Median length of stay, days (IQR)	21 (8–38)	23 (9–50)	19 (8–33)	<0.02

*LFNC* low-flow nasal cannula, *HFNC* high-flow nasal cannula.

*Estimated from *χ*^2^ or Wilcoxon rank sums test, respectively.

In the post-intervention era, 29/218 (13.3%) met bundle criteria at least once. The median (IQR) gestational age was 32.0 (24.7–38.7); birth weight was 1.9 kg (0.8–3.0); sex was 16/29 (57.1%) male; 11 were black, 10 white, 5 Hispanic, and 3 unknown. The postnatal age at bundle implementation was 21 days (8–51). The discharge disposition was 16 home, 9 transferred to another unit, and 4 died.

### Primary outcomes (ventilation – free days)

**Fig. 3:**
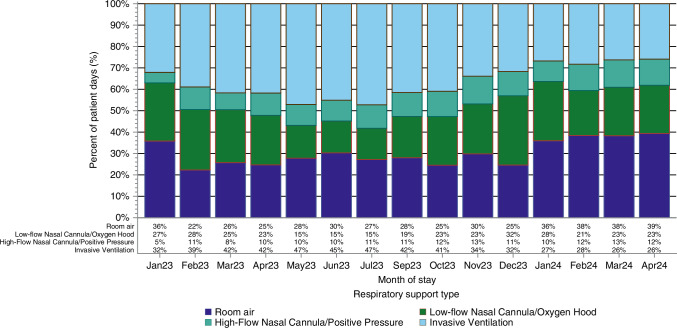
Comparison of the percentage of days on each type of respiratory support by month.

**Fig. 4:**
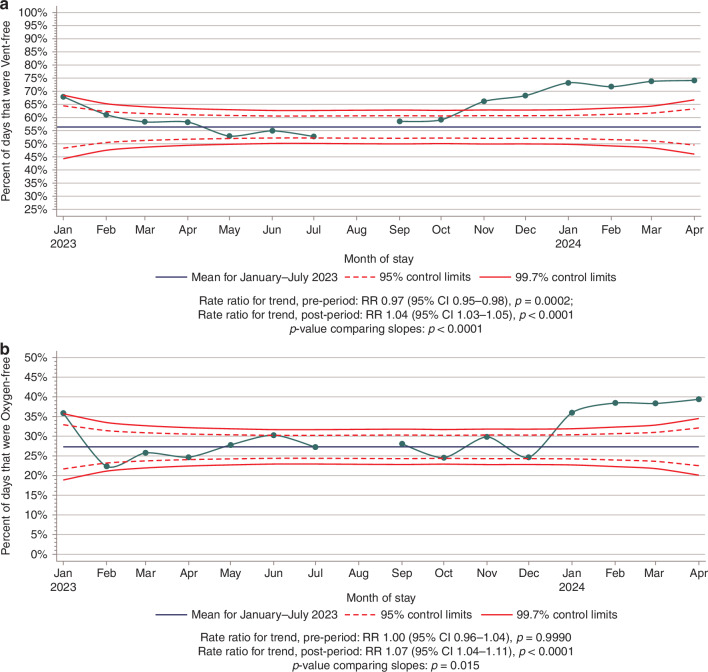
Run charts evaluating outcomes show special cause variation improvements that coincide with bundle implementation. **a** Shows the percentage of ventilator – free days by month before and after the intervention (August). **b** Shows the percentage of oxygen – free days by month before and after the intervention (August).

A monthly trend of percentage of patient days spent on room air, nasal cannula, non-invasive ventilation, and invasive ventilation is shown in Fig. 3. Overall, there was a decrease in percentage of days spent on invasive ventilation and an increase in percentage of days spent on room air (*p* < 0.01). Figure 4a shows the percentage of ventilator-free days by month with a special cause variation showing a statistically significant difference in the percentage of ventilator-free days after bundle implementation (*p* < 0.01). Table 3 shows that the percentage of ventilator-free days was higher in the post-era compared to the pre-era (5592/8335 (67%)) vs. (3732/6619 (56%); *p* < 0.001). Each month during the pre-bundle period, the rate of ventilator-free days decreased by an average of 3.3% (MPC −3.3, 95% CI −5.0, −1.6; *p* = 0.0002). Comparatively, the ventilator-free days increased 4% per month in the post-implementation group (MPC 4.0, 95% CI 2.7, 5.3; *p* < 0.0001).

**Table 3:** Monthly percent changes* (MPCs) with associated 95% confidence intervals (CIs) for the comparison of the rates of ventilator- and oxygen-free days between periods of bundle implementation

	Pre-bundle (Jan 2023–July 2023)		Post-bundle (September 2023–April 2024)		Difference between eras
	Days/Total	MPC (95% CI)	Days/Total	MPC (95% CI)	*p*-value
Ventilator-free	$\frac{{{\mathrm{3732}}}}{{{\mathrm{6619}}}}\,(56 \%)$	−3.3 (−5.0, −1.6) *p* = 0.0002	$\frac{{{\mathrm{5592}}}}{{{\mathrm{8335}}}}\,(67 \% )$	4.0 (2.7, 5.3) *p* < 0.0001	*p* < 0.0001
Oxygen-free	$\frac{{{\mathrm{1807}}}}{{{\mathrm{6619}}}}\,(27 \%)$	0.0 (−0.04, 4.5) *p* = 0.9990	$\frac{{{\mathrm{2603}}}}{{{\mathrm{8335}}}}\,(31 \% )$	7.1 (3.5, 10.7) *p* < 0.0001	*p* = 0.0150

* Estimated from a negative binomial regression using patient days within a given month as the model offset.

### Secondary outcomes

Statistically significant special cause variation was detected in the mean percentage of oxygen-free days per month in January 2024 (Fig. 4b). The percentage of oxygen-free days was higher in the post-era compared to the pre-era (2603/8335 (31%)) vs. 1807/6619 (27%); *p* < 0.015. In the post-bundle era, the oxygen-free days increased by 7.1% per month (MPC 7.1, 95% CI 3.5, 10.7; *p* < 0.0001). After protocol implementation, the median length of stay was lower (23 days (IQR 9–50) vs. 19 days (IQR 8–33), *p* < 0.02). We did not find differences in mortality rates between eras.

### Process measures

Table 4 lists reasons for bundle entry and implementation rates of each bundle element during the post-intervention era. Median length of bundle duration was 9 days (IQR 6.8–14). The most common reason for bundle entry was fluid overload (60.7%), followed by major surgery (42.9%), sepsis (35.7%), pressor support for hypotension (28.6%), AKI by SCr (21.4%), NEC (14.3%), and SIP (7.1%). Bundle elements were separated into daily events and one-time events. Fluid balance and total fluid intake (TFI) goals, along with nephrotoxic medication review, were discussed with high frequency: 88.8%, 82.7%, and 83.7%, respectively. Mean arterial pressure goals were documented less often (56.2%). Only five patients (17.9%) had a formal documented furosemide stress test performed, although diuretics were prescribed on half of the patient days reviewed (50.8%). Of the one-time bundle events, steroids were utilized for hypotension in nine patients (32.1%), and renal ultrasound was obtained in eight patients (28.6%). Less commonly performed components included performing an abdominal tap (3.6%), ultrafiltration (3.6%), and obtaining a uric acid level (10.7%). No patients had hyperuricemia. No bladder pressures were measured. Blood albumin levels <2.5 g/dL were detected 51 times, and 18/51 (35.3%) received 25% albumin infusions.

**Table 4:** Frequency of bundle inclusion criteria and compliance for one-time and daily bundle events

BUNDLE CRITERIA MET, *n* (%)	
Fluid overload	17 (60.7)
Sepsis	10 (35.7)
Pressor use	8 (28.6)
Major surgery	12 (42.9)
AKI by serum creatinine	6 (21.4)
NEC	4 (14.3)
SIP	2 (7.14)
**ONE-TIME BUNDLE EVENTS,** ***n*** **(%)**	*N* = 28 patients
Lasix stress test performed, *n* (%)	5 (17.9)
Uric acid obtained	3 (10.7)
RUS obtained	8 (28.6)
Rasburicase given if uric acid >9	0 (0)
Abdominal tap is performed if indicated	1 (3.6)
Steroids are used for hypotension	9 (32.1)
Ultrafiltration performed	1 (3.6)
Foley utilized	8 (28.6)
25% albumin administered if albumin <2.5	18 (35.3)*
Bladder pressure measured	0 (0)
**DAILY BUNDLE EVENTS,** ***n*** **(%)**	*N* = 312 days
Fluid balance discussed	278 (88.8)
Nephrotoxic medications reviewed	262 (83.7)
MAP goals discussed	176 (56.2)
TFI goals discussed	259 (82.7)
Diuretics given for FO	159 (50.8)

*Differing denominator of 51 for the amount of albumin levels <2.5 g/dL.

## Discussion

In this quality improvement study designed to prevent and mitigate fluid overload, we found an increase in ventilator-free days, oxygen-free days and a reduction in the NICU length of stay after CAN-U-P-LOTS bundle implementation. This suggests that a systematic protocolized approach designed to limit fluid overload may improve important clinical outcomes in high-risk infants. Development and execution of this clinical bundle encompass strategies that are available in most level IV NICUs and are used by many providers. A main feature of this initiative was the identification of high-risk neonates and systematic assessments/interventions early in the course of critical illness.

Interestingly, a relatively small percentage of infants met criteria for bundle inclusion (13%), yet there was still a significant increase in ventilator-free and oxygen-free days in the post-implementation cohort. A potential explanation is that implementation of the bundle led to changes in clinical practice that limited the number of neonates who needed to enter the bundle for fluid overload and/or AKI. The high compliance rates of fluid balance assessments and of total fluid intake evaluation support this explanation. The impact of education in quality improvement studies is known to have far-reaching effects beyond the specific population in quality improvement studies. Specifically, this has been evident in other pediatric studies to prevent nephrotoxicity in children^31^ and in quality improvement studies on AKI recognition.^32^ Further explanations for improved clinical practices that spill over to other populations include system-wide changes, enhanced provider skills, and creation of a learning culture.

To our knowledge, this quality improvement study was the first to evaluate how a protocolized bundle in high-risk critically ill infants is associated with a reduction in clinically meaningful negative effects of fluid overload. Previous studies have shown similar findings in other critically ill populations. For example, Diaz et al. showed that in children with ARDS (acute respiratory distress syndrome) and sepsis, a judicious fluid strategy reduced the peak fluid balance, mechanical ventilation, and PICU length of stay.^15^ In 2023, Goldstein et al. demonstrated that a pathway that uses a risk stratification score plus a urinary biomarker that directs fluid delivery and nephrology consultation led to decreased time on mechanical ventilation, CRRT, and PICU length of stay.^16^ In children admitted from the emergency department for pediatric septic shock, Akcan Arikan et al. showed how a bundle designed to mitigate fluid overload decreased AKI, ICU lengths of stay, and mortality.^33^ Our study differs in the patient age, bundle indications, bundle specifics, and primary outcomes. Collectively, these studies suggest that protocols to prevent and treat fluid overload in high-risk patients may improve important clinical outcomes.

The study strengths include ample provider education using a clear management bundle that was developed through multidisciplinary consensus based on evidence from the medical literature. All therapies in the bundle are available for use in our hospital. Despite these strengths, we recognize several limitations to the study, including short duration, single-center study, low sample size, and non-electronic tracking. We acknowledge that we may not have captured long-term trends and outcomes. We also acknowledge that we do not have fluid balance data for patients entered into the bundle, given the study design. Although the team was instructed to screen every patient every day for criteria into the bundle, we cannot assure that patients were evaluated every day. In addition, providers were aware of our study interventions and goals, which may have led to modifications in other aspects of care. However, the sustained reduction in mechanical ventilation in the current study suggests that differences were unlikely to be explained by demand characteristics or novelty effects. Furthermore, following feedback after the conclusion of the study, neonatal providers identified that paper tracking may have been a barrier, as papers can get lost or misplaced, thus the number of actual bundles and the interventions performed may be lower than described. Lastly, we recognize that this small study did not have the granularity to determine the exact intervention/ interventions that were most important to the observations being made. In fact, it is possible that the differences between eras could be due to the Hawthorne effect, whereby simply being vigilant and mindful to prevent fluid excess (and not one specific intervention) may be the most important etiology for better outcomes.

In conclusion, our quality improvement study suggests that our approach of identifying high-risk patients and implementing strategies using the CAN-U-P-LOTS bundle to mitigate and prevent fluid overload can improve important clinical outcomes in critically ill infants. Further research is needed to better understand the impact of each of the bundle elements and develop strategies to optimize the implementation of all bundle elements into clinical workflows. In addition, we note the importance of tracking intake, output, weight, and calculations for fluid balance on daily rounds in critically ill neonates to assist the team and in future quality improvement studies. Strategies to determine how to best track information and optimize compliance for all bundle elements are needed. Ultimately, a multi-center clustered randomized clinical trial is needed to corroborate our findings, provide external validity, and address some of the limitations outlined above.

### Data statement

This study was conducted in accordance with relevant guidelines and regulations.

## Data Availability

The data that support the findings of this study are available from Lindsey Gordon upon request.

## Code availability

The code used to analyze the data is available from Russell Griffin upon request.
